# African Under-Utilized Medicinal Leafy Vegetables Studied by Microtiter Plate Assays and High-Performance Thin-Layer Chromatography–Planar Assays

**DOI:** 10.3390/molecules29030733

**Published:** 2024-02-05

**Authors:** Ibukun O. Oresanya, Ilkay Erdogan Orhan, Julia Heil, Gertrud E. Morlock

**Affiliations:** 1 Department of Pharmacognosy, Faculty of Pharmacy, Gazi University, Emniyet, Taç Sokağı No. 3, Yenimahalle, Ankara 06330, Turkey; ayoola.ibukun@gmail.com (I.O.O.); iorhan@gazi.edu.tr (I.E.O.); 2 Chair of Food Science, Institute of Nutritional Science, Justus Liebig University Giessen, Heinrich-Buff-Ring 26-32, 35392 Giessen, Germany; julia.heil@ernaehrung.uni-giessen.de; 3 Center for Sustainable Food Systems, Justus Liebig University Giessen, Senckenbergstr. 3, 35390 Giessen, Germany

**Keywords:** ethnomedicine, antidiabetic, antimicrobial, antioxidant, anticholinesterase, planar bioassays, planar chromatography, electrospray ionization high-resolution mass spectrometry

## Abstract

Biological activities of six under-utilized medicinal leafy vegetable plants indigenous to Africa, *i.e*., *Basella alba*, *Crassocephalum rubens*, *Gnetum africanum*, *Launaea taraxacifolia, Solanecio biafrae*, and *Solanum macrocarpon*, were investigated via two independent techniques. The total phenolic content (TPC) was determined, and six microtiter plate assays were applied after extraction and fractionation. Three were antioxidant *in vitro* assays, *i.e*., ferric reducing antioxidant power (FRAP), cupric reduction antioxidant capacity (CUPRAC), and 2,2-diphenyl-1-picrylhydrazyl (DPPH) scavenging, and the others were enzyme (acetylcholinesterase, butyrylcholinesterase, and tyrosinase) inhibition assays. The highest TPC and antioxidant activity from all the methods were obtained from polar and medium polar fractions of *C. rubens, S. biafrae,* and *S. macrocarpon*. The highest acetyl- and butyrylcholinesterase inhibition was exhibited by polar fractions of *S. biafrae*, *C. rubens,* and *L. taraxacifolia*, the latter comparable to galantamine. The highest tyrosinase inhibition was observed in the *n*-butanol fraction of *C. rubens* and ethyl acetate fraction of *S. biafrae. In vitro* assay results of the different extracts and fractions were mostly in agreement with the bioactivity profiling via high-performance thin-layer chromatography–multi-imaging–effect-directed analysis, exploiting nine different planar assays. Several separated compounds of the plant extracts showed antioxidant, *α-*glucosidase, *α-*amylase, acetyl- and butyrylcholinesterase-inhibiting, Gram-positive/-negative antimicrobial, cytotoxic, and genotoxic activities. A prominent apolar bioactive compound zone was tentatively assigned to fatty acids, in particular linolenic acid, via electrospray ionization high-resolution mass spectrometry. The detected antioxidant, antimicrobial, antidiabetic, anticholinesterase, cytotoxic, and genotoxic potentials of these vegetable plants, in particular *C. rubens, S. biafrae*, and *S. macrocarpon*, may validate some of their ethnomedicinal uses.

## 1. Introduction

Plants comprise numerous chemicals with potential for adjuvant treatment of diseases and have played unique and leading roles in drug research. Since phytochemicals from African flora in particular are still untapped [1,2], some under-utilized medicinal leafy vegetables grown in West Africa were investigated. The six plants in focus were *Launaea taraxacifolia* (Willd.) Amin ex C. Jeffrey, *Crassocephalum rubens* (Juss. ex Jacq.) S. Moore, and *Solanecio biafrae* (Oliv. & Hiern) C. Jeffrey from the family Asteraceae, as well as *Solanum macrocarpon* L., *Basella alba* L., and *Gnetum africanum* Welw. from the families Solanaceae, Basellaceae, and Gnetaceae, respectively. These uncommon vegetable types of medicinal plants are only available in local markets and rural areas [3,4], which explains the limited research data available. They are used traditionally for the treatment of diabetes, body pains, wounds, arthritis, fever, cancer, ulcer, and epilepsy and have been reported to display antioxidant, antimicrobial, anti-inflammatory, anticancer, wound healing, and antiulcer activities [5,6,7,8,9,10].

Bioactivity-guided drug discovery approaches have been used successfully to identify important drug leads especially from medicinal plants [11]. *In vitro* biological assays using 96- or 384-well microtiter plates and chromatographic fractionation (bioassay-guided fractionation) have been applied in medicinal plant research to screen for biological activities and to identify the responsible molecules [12]. Combining a cholinesterase inhibitor with an antioxidant to alleviate oxidative stress is a targeted strategy for the treatment of Alzheimer’s disease (AD) [13,14,15]. Hence, the inhibition of acetylcholinesterase (AChE) and butyrylcholinesterase (BChE), which cleave the neurotransmitters acetylcholine and butyrylcholine into choline and respective acetic acid and butyric acid, is of interest. Since the accumulation of metals (iron, copper, lead, zinc, aluminum, cadmium, etc.) has a significant impact on its pathogenesis [16], antioxidant potentials of plant extracts, *e.g*., via the 2,2-diphenyl-1-picrylhydrazyl (DPPH•) scavenging assay, were also studied to inhibit the production of reactive oxygen species by metals such as iron and copper. Tyrosinase (TYR) inhibition has become a recent target in Parkinson’s disease research [17], since TYR in excess amount (a copper-containing enzyme involved in the synthesis of melanin and neuromelanin formation) causes deterioration in functions of nigral neurons [18].

In contrast to *in vitro* assays, planar assays directly point to bioactive compound zones due to the integrated separation. High-performance thin-layer chromatography–multi-imaging (HPTLC–ultraviolet/visible/fluorescence detection, UV/Vis/FLD) combined with effect-directed analysis (EDA) has been proven as a straightforward and efficient tool in the screening for active phytochemicals, among hundreds to thousands of phytochemicals present in complex plant samples [19]. Its hyphenation to high-resolution mass spectrometry (HRMS) is highly targeted for further characterization of the detected bioactive compounds [20,21,22]. Thus, the mentioned assays were also performed as planar assays. In addition, α-amylase, α- glucosidase, and antimicrobial assays (Gram-positive *Bacillus subtilis* and Gram-negative *Aliivibrio fischeri*) were applied to identify antidiabetic, anti-infective, and wound-healing properties of plant extracts [19,20] as well as cytotoxicity and genotoxicity assays [23] (the latter also with S9 liver metabolization [24]) since it is important information for the traditional use of medicinal plants.

This study aimed to detect and evaluate the biological activities and responsible bioactive compounds of six African under-utilized medicinal leafy vegetables, *i.e*., *Launaea* taraxacifolia (Willd.) Amin ex C. Jeffrey, *Crassocephalum rubens* (Juss. ex Jacq.) S.Moore, *Solanecio biafrae* (Oliv. & Hiern) C. Jeffrey, *Basella alba* L.*, Solanum macrocarpon* L., and *Gnetum africanum* Welw. Two independent techniques (*in vitro* assays and planar assays) were applied to explain part of their traditional uses. Therefore, their total phenolic content (TPC) was determined and six spectrophotometric *in vitro* microtiter plate assays were applied and compared with each other. Among these were three antioxidant assays, *i.e*., ferric reducing antioxidant power (FRAP), cupric reduction antioxidant capacity (CUPRAC), and 2,2-diphenyl-1-picrylhydrazyl (DPPH•) scavenging, and three enzyme inhibition assays, *i.e*., AChE, BChE, and TYR inhibition assays. The results were compared to the HPTLC–UV/Vis/FLD–EDA profiling exploiting nine assays (*i.e*., DPPH• scavenging, AChE, BChE, *α-*amylase, and α-glucosidase inhibition, *Aliivibrio fischeri*, *Bacillus subtilis*, cytotoxicity, and genotoxicity assays). Prominent bioactive compound zones were further characterized by heated electrospray ionization high-resolution mass spectrometry (HPTLC–UV/Vis/FLD–HESI-HRMS).

## 2. Results and Discussion

### 2.1. TPC As Well As FRAP, CUPRAC, and DPPH• Scavenging Antioxidant In Vitro Assays

The TPC of each extract/fraction was determined via the Folin–Ciocalteu method [25] and calculated as gallic acid equivalents (GAE) per gram of extract dry weight. The FRAP of the extracts and the reference quercetin was measured based on the reduction of Fe(III) to Fe(II) at low pH leading to the formation of a colored Fe (II)-tripyridyltriazine complex [26]. Via the CUPRAC assay, antioxidants in the extracts/fractions and the reference gallic acid reduced the copper–neocuproin complex [Cu (II)-Nc] to a highly colored Cu (I)-Nc, which was measured [27]. The antioxidant activity of the extracts/fractions and quercetin, the reference, was also measured by their ability to scavenge the stable DPPH• radical. Their half maximal effect or inhibition concentrations (IC_50_) were calculated.

The TPC (Table 1) varied across the studied extracts/fractions with the highest TPC for *n*-butanol (40.2 mg GAE/g) and ethyl acetate (38.7 mg GAE/g) fractions of *C. rubens*, followed by *S. macrocarpon n*-butanol (34.9 mg GAE/g), and *S. biafrae* ethyl acetate fractions (31.0 mg GAE/g). The TPC was similar to the *in vitro* FRAP and CUPRAC assay results. For the *in vitro* DPPH• scavenging assay, the ethyl acetate fraction of *C. rubens* exhibited the highest activity (87.2%, IC_50_ 27.7 µg/mL), followed by its *n*-butanol fraction (84.7%, IC_50_ 47.7 µg/mL) as well as the ethyl acetate fraction of *S. biafrae* (84.4%, IC_50_ 62.8 µg/mL) as well as the ethyl acetate fraction (84.9%, 73.5 µg/mL) and ethanol extract (78.9%, IC_50_ 62.7 µg/mL) of *S. macrocarpon*. These three plants (*C. rubens*, *S. biafrae*, and *S. macrocarpon*) exhibited among the six plants a noteworthy antioxidant activity with results almost comparable to that of the reference quercetin (89.5%). Yusuff et al. [28] compared the DPPH• scavenging activity of only methanol extracts of *S. macrocarpon* and *C. rubens*, and reported that *S. macrocarpon* methanol extract had higher antioxidant activity than that of *C. rubens*. Although this is similar to our ethanol extract results, ethyl acetate and *n*-butanol fractions of *C. rubens* led to a higher activity than other fractions of *S. macrocarpon* (Table 1). From Pearson’s correlation coefficient analysis, the antioxidant activity of all plant extracts/fractions showed a strong positive correlation between TPC and FRAP (r^2^ = 0.90), TPC and DPPH• (r^2^ = 0.79), TPC and CUPRAC (r^2^ = 0.75), FRAP and DPPH• (r^2^ = 0.87) as well as FRAP and CUPRAC (r^2^ = 0.70). Phenolic compounds are known for their antioxidant properties and other important pharmacological effects [29], which might be responsible for their antioxidant activity.

### 2.2. AChE, BChE, and TYR In Vitro Inhibition Assays

The potentials of the plant extracts/fractions to inhibit AChE, BChE, and TYR were evaluated. According to Ellman’s spectrophotometric method [30] modified by [31], thiol esters used as substrates are hydrolyzed by AChE or BChE to release thiocholine, which react with 5,5′-dithio-bis(2-nitrobenzoic) acid to form a yellow-colored 2-nitro-5-thiobenzoate. Inhibition of the enzymes is measured as lighter coloration. The results indicated a better BChE than AChE inhibition (Table 2). The highest BChE inhibition was given by the water extract of *L. taraxacifolia* (66.4%, IC_50_ 114.0 µg/mL), which was close to the reference galantamine (69.6%). The water extract (52.6%, IC_50_ 183.4 µg/mL) and methanol–water fraction of *S. biafrae* (58.8%, IC_50_ 135.3 µg/mL), the ethanol extract of *B. alba* (53.0%, IC_50_ 121.5 µg/mL), and the methanol–water fraction of *C. rubens* (52.0%, IC_50_ 176.8 µg/mL) followed. Only the methanol–water fraction of *S. biafrae* showed moderate inhibition of AChE (53.6%, IC_50_ 207.0 µg/mL). For tyrosinase inhibition, only the *n*-butanol fraction of *C. rubens* (42.9%) and ethyl acetate fraction of *S. biafrae* (37.4%) displayed a noteworthy inhibition. These results indicated that both samples contain some polar compounds that are able to inhibit these enzymes, but comparatively lower than the reference α-kojic acid (87.5%).

Only a few reports exist in the literature on the potentials of these plants to alleviate neurodegenerative diseases. Adedayo [32] reported AChE and BChE inhibition by phenolic-rich *L. taraxacifolia* extracts. Oboh et al. [33] studied the efficacy of dietary inclusion of *C. rubens,* a closely related species to *C. rubens* on the improvement of memory indices in fruit fly (*Drosophila melanogaster*) Alzheimer’s disease model. The result revealed that addition of the leaf powder of *C. crepidioides*, a close species to *C. rubens,* in the fruit fly feed improved survival rate and behavioral performance by 70–80% and also significantly inhibited AChE, BChE, and monoamine oxidase (MAO). Phenolic extract of *S. biafrae* was reported to inhibit both AChE and BChE (Ajiboye et al. [34]), which is consistent with our result, where *S. biafrae* aqueous methanol fraction solely inhibited both AChE and BChE. The neuroprotective effects of *S. biafrae* may be attributed to the phenolic compounds (gallic acid, chlorogenic, caffeic acid, rutin, quercetin, and kaempferol) identified in the extract [34,35].

### 2.3. Effect-Directed Profiling via Nine Different Planar Assays by HPTLC–UV/Vis/FLD–EDA

**Figure 1:**
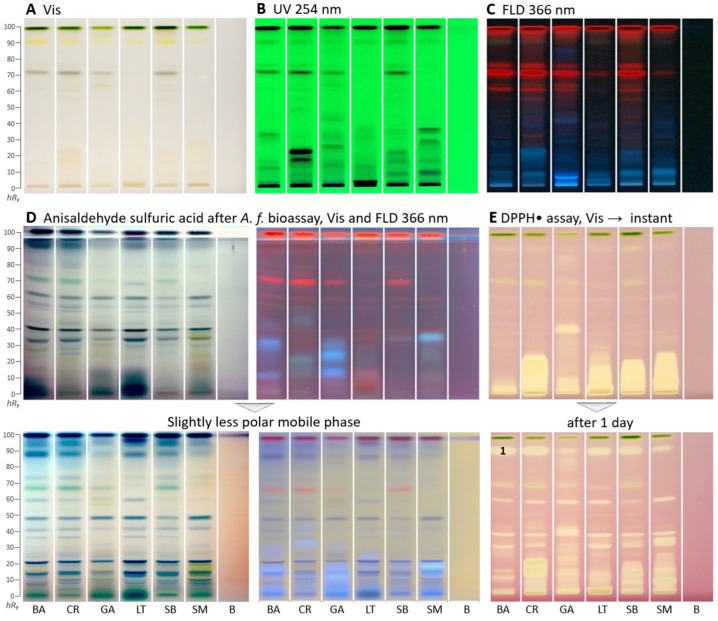
HPTLC fingerprints (**A**–**D**) and radical scavenging autogram (**E**) of *Basella alba* (BA), *Crassocephalum rubens* (CR), *Gnetum africanum* (GA), *Launaea taraxacifolia* (LT), *Solanecio biafrae* (SB), and *Solanum macrocarpon* (SM) along with solvent blank (**B**), all 5 µL/band, developed on HPTLC plates silica gel 60 F_254_ with ethyl acetate–toluene–methanol–water (4:1:1:0.4, *V/V/V/V*) and detected under (**A**) white light illumination (Vis), (**B**) UV 254 nm, and (**C**) FLD 366 nm as well as (**D**) white light illumination and FLD 366 nm after the *Aliivibrio fischeri* bioassay, followed by derivatization with the anisaldehyde sulfuric acid reagent; also shown with a slightly less polar mobile phase in the ratios (4:1:0.75:0.375, *V/V/V/V;* color changes due to reagent stored too long) and (**E**) white light illumination after the DPPH• assay (only 3 µL/band applied) instantly and after 1 day; zone 1 marked was recorded by HRMS.

The bioactivity profiling was performed by HPTLC–UV/Vis/FLD–EDA for these plants for the first time. As extractant of each lyophilized plant powder, the mixture ethyl acetate–ethanol–water (1:1:1, *V/V/V*) was chosen because it has been proven in other studies [36,37] to be a good compromise across polarities to extract most compounds at a time in order to avoid the preparation of so many extracts as prepared for the *in vitro* assays for fractionation of the many compounds present. Such mixtures are not so commonly used, but very effective for bioactivity screening in terms of more sustainable analytical chemistry workflows. However, the extraction of comparatively more apolar compounds (ethyl acetate component) can cause solubility problems for buffered, highly polar *in vitro* assays. In contrast, planar assays do not have such solubility issues. As a mobile phase, the solvent mixture consisting of ethyl acetate–toluene–methanol–water (4:1:1:0.4, *V/V/V/V*) was used. This mobile phase originated from a previous study on botanicals Schreiner et al. [21] (ethyl acetate–toluene–formic acid–water 16:4:3:2, *V/V/V/V*), but substituting formic acid by methanol and adjusting the ratios of the two polar solvent components. In the microtiter plate assay (Table 1), *C. rubens, S. biafrae*, and *S. macrocarpon* had the highest TPC. This was similar to the presence of many strong absorbing zones (most likely phenolic compounds among others) in the HPTLC–UV chromatogram detected at 254 nm (Figure 1B), wherein *C. rubens* showed the most intense zones followed by *S. biafrae* and *S. macrocarpon*. In the HPTLC chromatogram at FLD 366 nm (Figure 1C), coumarins and other phenolic compounds, often observed as blue fluorescent zones, were present in all samples along with red fluorescent chlorophyll zones.

As a reagent/detection sequence (Figure 1D), the derivatization with anisaldehyde sulfuric acid reagent even worked on the bioautogram after performance of the *Aliivibrio fischeri* bioassay, discussed later. This more universal derivatization reagent revealed organic compounds such as steroids and terpenes present in the samples as violet, blue, and gray colored zones. When the anisaldehyde sulfuric acid reagent was stored too long (*e.g*., for a year), the background color turned rose under white light illumination and lightened at FLD 366 nm. Thus, it had to be prepared freshly depending on the frequency of its use.

In the DPPH^•^-Vis autogram (Figure 1E), antioxidant compounds appeared as yellow zones against a purple background. Polar antioxidant compounds were detected in all samples, especially with strong intensity in *C. rubens, S. macrocarpon*, and *S. biafrae*. Intense zones were observed close to the start zone (*hR*_F_ 0–20), indicating polar antioxidant compounds. These results are in accordance with the antioxidant *in vitro* microtiter assay results, where *C. rubens, S. macrocarpon*, and *S. biafrae* had the highest antioxidant activity (Table 1). In the microtiter plate assay, the ethyl acetate and *n*-butanol fractions had the highest antioxidant activity, which is supported by the intense antioxidant zones detected at the low *hR*_F_ range in the HPTLC–DPPH^•^–Vis autogram. To detect time-dependent changes in the antioxidative profile and thus antioxidant reactions over time, the same autogram was recorded after one day. As a result, it expressed more differentiated antioxidative zones (Figure 1E), strong ones got weaker and minor ones got stronger. This showed that time-dependent changes and the antioxidant reactivity rate over time can be studied via the planar DPPH^•^ assay. Such differentiated information cannot be obtained by the corresponding *in vitro* assay, providing only a sum value.

**Figure 2:**
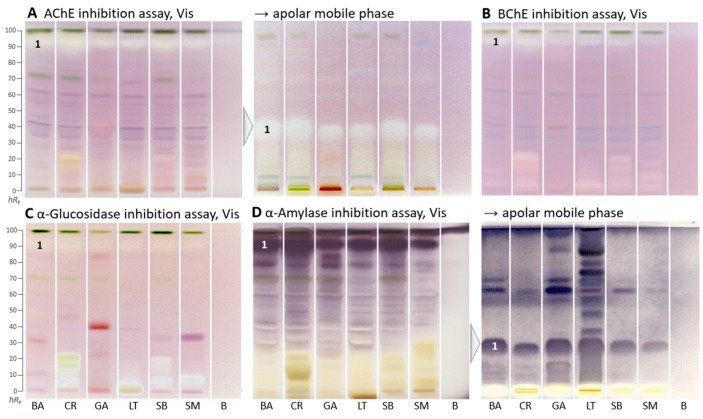
HPTLC–enzyme inhibition–Vis autograms of *Basella alba* (BA), *Crassocephalum rubens* (CR), *Gnetum africanum* (GA), *Launaea taraxacifolia* (LT), *Solanecio biafrae* (SB), and *Solanum macrocarpon* (SM) along with solvent blank (**B**), all 5 µL/band, developed on HPTLC plates silica gel 60 F_254_ with ethyl acetate–toluene–methanol–water (4:1:1:0.4, *V/V/V/V*), or apolar mobile phase toluene–ethyl acetate (7:3, *V/V*), and detected under white light illumination (Vis) after the (**A**) AChE, (**B**) BChE, (**C**) α-glucosidase, and (**D**) α-amylase assays; zone 1 marked was recorded by HRMS.

The following four enzyme assays revealed inhibiting compounds as colorless white zones on the respective purple background (Figure 2A–C) or as purple zones on a white background (Figure 2D). Both HPTLC–AChE/BChE inhibition–Vis autograms (Figure 2A,B) showed a similar compound pattern. The most prominent colorless inhibition zone was at *hR*_F_ 90 (zone 1) and inhibited both AChE and BChE in all samples. In contrast to the respective *in vitro* assays, the inhibition was stronger for AChE than BChE. However, colored compounds, which mitigate/suppress the inhibition signal of *in vitro* assays (providing only a mixed sum value), were clearly separated here. The AChE inhibition zone 1, which was close to the solvent front, was eluted to only *hR*_F_ 35 using an apolar mobile phase and thus better detectable. White inhibition zones, which remained at the start zone, were comparatively more prominent in the BChE inhibition autogram (Figure 2B).

Similar AChE and BChE inhibition zones (*hR_F_* 15 and 20) were seen in *C. rubens* and *S. biafrae*, though weaker in *S. biafrae* (Figure 2B). This similar compound pattern can be explained by their phytochemical similarity since both are from the family Asteraceae, although *L. taraxacifolia* from the same plant family showed a different compound pattern. The visually evaluated sum of all inhibition zones in the BChE autogram (Figure 2B) decreased in intensity from *C. rubens*, *L. taraxacifolia*, *S. biafrae* and *S. macrocarpon*, which was consistent with the respective *in vitro* microtiter plate assay result, where the water extract of *L. taraxacifolia* (66.4%, IC_50_ 114.0 µg/mL) and aqueous methanol fractions of *S. biafrae* (58.8%, IC_50_ 135.3 µg/mL) and *C. rubens* (52.0%, IC_50_ 176.8 µg/mL) exhibited highest BChE inhibition. Differences in the order can be explained by the different extraction system (a compromise mixture of solvents across polarities) for the planar bioassays.

Respective autograms for the antidiabetic activity revealed the presence of α-glucosidase- (Figure 2C) and α-amylase-inhibiting compounds (Figure 2D) in all plant samples. Regarding the α-glucosidase inhibition, *C. rubens, L. taraxacifolia*, *S. biafrae*, and *S. macrocarpon* showed comparatively strong polar inhibition zones at *hR*_F_ 0–20 (Figure 2C), similar, though much stronger, in pattern to the previous BChE assay. Regarding the α-amylase inhibition, a prominent zone was evident at *hR*_F_ 90 (Figure 2D, zone 1). It was assumed to be the same compound, which showed antioxidative activity (after 1 day) and AChE/BChE inhibition as discussed, due to the similar horizontal pattern of zone 1 across all samples. It showed that these vegetable plants contain compounds with strong α-amylase and α-glucosidase inhibitory activity. Inhibition of these enzymes, which delays the breakdown of saccharides leading to reduction in the rate of glucose absorption and lowering the postprandial serum glucose level, is a key mechanism in the management of diabetes mellitus [38]. Hence, this outcome underlined the traditional use of these leafy vegetables (*S. biafrae*, *C. rubens,* and *L. taraxacifolia*) for the treatment of diabetes in West-Africa [9,39,40]. Our results are also in agreement with in vivo and *in vitro* studies about their antidiabetic activity. Ayoola et al. [41] and Ajiboye et al. [42] reported the antihyperglycemic activity of *S. biafrae* determined from in vivo alloxan-induced hyperglycemic experiments using rats. Ajiboye et al. [34] reported α-amylase (IC_50_ 126.9 µg/mL) and α-glucosidase (IC_50_ 139.7 µg/mL) inhibition by phenolic extract of *S. biafrae*. Oyebode et al. [43] reported α-glucosidase and lipase inhibition by *C. rubens* ethyl acetate and aqueous extracts, where *C. rubens* extracts also inhibited intestinal glucose absorption in ex vivo studies by the same group with results comparable to controls. Gbadamosi et al. [44] and Adjei et al. [45] reported the antidiabetic activity of *L. taraxacifolia* through alloxan- and streptozotocin-induced diabetic rat models, respectively.

**Figure 3:**
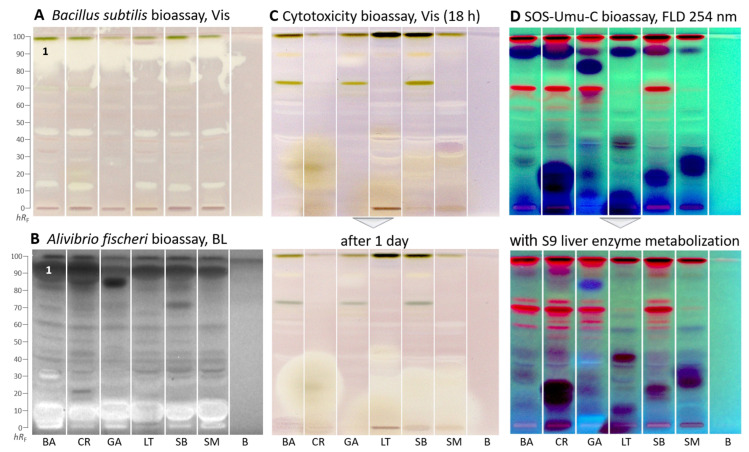
HPTLC–biological assays–Vis/BL/FLD bioautograms of *Basella alba* (BA), *Crassocephalum rubens* (CR), *Gnetum africanum* (GA), *Launaea taraxacifolia* (LT), *Solanecio biafrae* (SB), and *Solanum macrocarpon* (SM) along with solvent blank (**B**), all 5 µL/band (except 15 µL for SOS-Umu-C and cytotoxicity bioassays), developed on HPTLC plates silica gel 60 F_254_ (for C/D on HPTLC plates silica gel 60) with ethyl acetate–toluene–methanol–water (4:1:1:0.4, *V/V/V/V*) and detected after the (**A**) *Bacillus subtilis* bioassay under white light illumination (Vis), (**B**) *Aliivibrio fischeri* bioassay as bioluminescence (BL) depicted as greyscale image, (**C**) cytotoxicity bioassay using the *Salmonella* Typhimurium cells with thiazol blue tetrazolium bromide substrate under white light illumination, and (**D**) SOS-Umu-C genotoxicity bioassay with fluorescein-digalactoside substrate, and for comparison, on a separate plate, the same with metabolization via the S9 liver enzyme system detected at FLD 254 nm; zone 1 (marked) was recorded by HRMS.

Apart from enzyme assays, biological suspension cell assays were used. In the Gram-positive *Bacillus subtilis* bioautogram (Figure 3A), antibacterial zones were detected as white zones on a purple background under white light illumination. In all samples, a very strong (already overloaded) antibacterial zone was detected at *hR*_F_ 90 (zone 1). Two further less prominent zones were at *hR*_F_ 14 and 45, though absent or weak in *G. africanum*.

In the Gram-negative *Aliivibrio fischeri* bioautogram (Figure 3B), bioactive zones were revealed as dark (lower energetic metabolism of the bacteria) or brightened zones (higher energetic metabolism) on the instantly bioluminescent plate background in all samples. The bacterial bioluminescence, depicted as greyscale image, was monitored for 30 min and revealed no substantial time-dependent changes. In all samples, one prominent zone was evident at *hR*_F_ 90 (zone 1) and another though weaker zone in the solvent front. The antibacterial zone pattern was different for *G. africanum,* which showed one intense antibacterial zone at *hR*_F_ 83. Another more intense zone at *hR*_F_ 70 was evident for *S. biafrae*. Further antibacterial zones were weaker. These results support other reports on the antimicrobial activity of the plants [46,47,48,49]. Brightened compound zones near the start zone indicated an increased energetic metabolism in the bacteria, which can be caused by saccharides or sugar alcohols or glycosides, etc.

In the cytotoxicity bioautogram (Figure 3C), using *Salmonella* Typhimurium cells incubating for 18 h and thiazol blue tetrazolium bromide as the substrate, few cytotoxic compounds were observed as colorless zones on a purple background. However, after one day, the polar cytotoxic zones (*hR*_F_ 0–20) of *C. rubens*, *L. taraxacifolia, S. biafrae*, and *S. macrocarpon* increased substantially, indicating an increase in cytotoxicity over time. In contrast, *G. africanum* showed only one weak cytotoxic compound zone at *hR*_F_ 83 (Figure 3C), which decreased over time. Only a few reports exist in the literature on the cytotoxic potentials of these species. Kumar et al. [50] reported the cytotoxicity effect of *B. alba* aqueous stem extract on HepG2 (hepatocellular carcinoma), A431 (epidermoid carcinoma), and MG63 (osteosarcoma) cell lines. Alhassan and Atawodi [51] investigated the effect of dietary inclusion of *C. rubens* on colon cancer induced through *N*-methyl-*N*-nitrosourea in Wistar rats. The results suggested that *C. rubens* could be effective in preventing the onset and progression of chemically induced colon carcinogenesis. Gnetin-C, a stilbene present in *G. africanum*, has been shown by some studies to possess potent anticancer property [52,53]. Koukoui et al. [54] reported the cytotoxic effect of *L. taraxacifolia* aqueous ethanol extract on HepG2 cells. According to Oluremi [55], *S. macrocarpon* extracts displayed potent cytotoxic activity against HeLa, HEp-2, and MCF-7 cancer cells.

In the genotoxicity bioautogram (Figure 3D), genotoxic substance zones were evident as the formed green fluorescent fluorescein end-product detected at FLD 254 nm [56]. Only a few weak genotoxic substance zones were detected at *hR*_F_ 40, 50, and 75 in several plants. However, prominent dark blue zones were observed indicating cytotoxicity. At these zone positions, the usually light green fluorescent background was not expressed due to cell death. The dark blue zones are in agreement with the previous cytotoxic compound zones. However, the fluorogenic fluorescein-digalactoside substrate used here is more sensitive in detection than the chromogenic thiazol blue tetrazolium bromide substrate used in the previous cytotoxicity bioassay, which explains the stronger response. It confirmed in particular the previous cytotoxicity results for *C. rubens*, *L. taraxacifolia, S. biafrae*, and *S. macrocarpon*. For these dark blue zones, genotoxicity cannot be excluded at lower amounts, and further dose–response studies are needed for clarification of the genotoxic potential.

Almost all genotoxic and cytotoxic compound responses were substantially reduced after on-surface detoxification via the S9 liver metabolization system (Figure 3D). The S9 liver metabolization system on HPTLC plates mimics the liver detoxification system of a healthy liver in the human body [24]. This indicated that the observed genotoxic and cytotoxic molecules can largely be detoxified in a healthy liver.

### 2.4. Comparison of Both Effect-Directed Analysis Techniques

For the first time, the results of *in vitro* microtiter plate assays were compared with those of the bioactivity profiling by HPTLC–UV/Vis/FLD–EDA for these plants. Generally, both methods are very useful in the drug discovery process. Microtiter plate assays are more commonly used for quantitatively evaluating biological activity [57]. However, IC_50_ values can also be calculated as well via HPTLC–UV/Vis/FLD–EDA, which is a more new area of research [56]. The used microtiter plate assays analyzed up to 96 samples at the same time, which gives room for the relevant dilutions and controls and it is cost effective. The solvent for dissolution of the plant extracts should not interfere or inhibit the enzyme reaction in the microtiter plate well. For solubility reasons, medium polar plant extracts are re-dissolved with low concentrations of dimethyl sulfoxide, ethanol, or methanol before *in vitro* assay screening [12]. In contrast, HPTLC–UV/Vis/FLD–EDA is compatible with any kind of extraction solvent, such as water, methanol, chloroform, ethyl acetate, *n*-hexane, etc., without affecting enzyme activity, since all solvents are evaporated before planar assay screening [58]. Otherwise, the same type of chemicals, buffers, and enzymes were used for both assay methods. For microtiter plate assays, a mixed sum value is obtained as result for a complex sample, therefore, it has to be used with caution since it is highly prone to signal interferences. For example, the presence of other colored compounds may counteract or suppress or cancel the measurement of the colorless inhibition signal. In contrast, the effect-directed HPTLC profiling can separate colorless inhibitors from interfering colored compounds, since the separation is integrated into planar assays. HPTLC–UV/Vis/FLD–EDA allows for the differentiation and identification of potential individual biochemical and biological active compounds. Their further characterization is possible by detection methods, such as HRMS and nuclear magnetic resonance spectroscopy [22]. HPTLC–UV/Vis/FLD–EDA is also cost-efficient (0.5–0.8 EUR/sample, depending on the enzyme costs) and fast (5–15 min/sample, depending on the incubation time of the assay) since up to 22 samples can be analyzed on one HPTLC plate. Recently, the open-source do-it-yourself 2LabsToGo system was introduced as a one-of-its-kind development in system engineering [59], which is highly sustainable regarding resources and has very low material costs to build and install the planar assay methodology. Its functionality was proven by various applications [60]. In contrast, the *in vitro* assay has to be followed by bioassay-guided fractionation and chromatographic analysis, which requires much more material resources and more time for analysis.

### 2.5. Characterization of the Bioactive Zone 1 via HRMS

**Figure 4:**
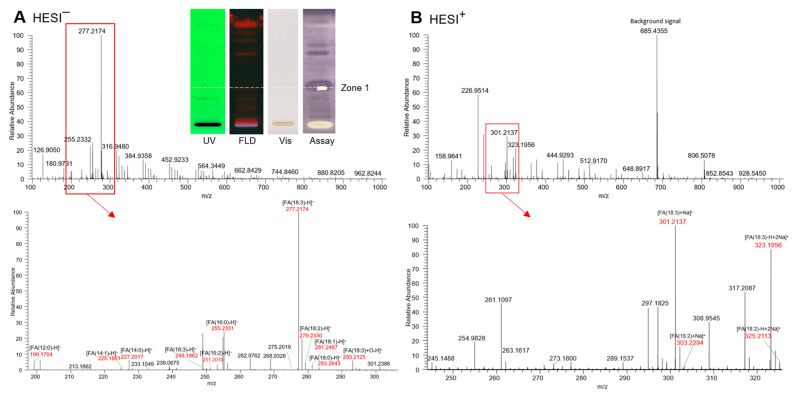
HPTLC–UV/Vis/FLD–HESI-HRMS spectra in the (**A**) negative and (**B**) positive ionization mode of the prominent multi-potent bioactive compound zone 1 (marked in Figure 1, Figure 2 and Figure 3) exemplarily recorded from *Basella alba* (5 µL/band applied and developed on silica gel 60 F_254_ HPTLC plate with toluene–ethyl acetate 7:3, *V/V*); post-HRMS performance of the α-amylase inhibition assay and respective autogram under white light illumination (Vis) as proof of the proper positioning on the active zone 1, which was originally not UV-active, not fluorescent and not visible.

The prominent apolar bioactive compound zone 1 was selected for further characterization via recording of HPTLC−HESI-HRMS spectra. The compound zone itself was not visible, not UV-active, and not fluorescent and thus not detectable in the initial UV/Vis/FLD chromatogram. First, in the bioprofiles, the zone was detected due to its versatile activities, such as DPPH• scavenging (Figure 1), AChE, BChE, α-glucosidase, and α-amylase inhibition (Figure 2) as well as activities against *Bacillus subtilis* and *Aliivibrio fischeri* (Figure 3). After application of exemplarily *Basella alba* and its separation using the apolar mobile phase system, the bioactive zone compound zone 1 was eluted from the HPTLC plate into the HESI–HRMS system using the open-source modified auto-TLC-LC-MS interface [61] and molecular formulae were tentatively assigned. After the zone elution, the respective α-amylase inhibition assay was performed to confirm the proper elution zone positioning, evident via the elution head imprint (Figure 4).

In the HESI^−^ mode, the deprotonated HRMS signal (base peak) at *m*/*z* 277.2174 [FA(18:3) − H]^−^ (Δ ppm −0.5) tentatively indicated linolenic acid (C_18_H_30_O_2_), which was confirmed by its sodium adduct at *m*/*z* 301.2137 [FA(18:3) + Na]^+^ (Δ ppm 0.5) and disodium adduct at *m*/*z* 323.1956 [FA(18:3) + 2Na − H]^+^ (Δ ppm −3.0) in the HESI^+^ mode. Oxidized linolenic acid (C_18_H_30_O_3_) was also evident at *m*/*z* 293.2152 [FA(18:3) + O − H]^−^ (Δ ppm −1.0). Although, with low signal intensity, it can be responsible for the antibacterial activity, cytotoxicity, and genotoxicity of samples [23]. As expected for the normal phase separation mechanism, further coeluting fatty acid signals, though much weaker, were revealed in the same zone and tentatively assigned to palmitic acid (C_16_H_32_O_2_) at *m*/*z* 255.2331 [FA(16:0) − H]^–^ (Δ ppm −0.7), linoleic acid (C_18_H_32_O_2_) at *m*/*z* 279.2330 [FA(18:2) − H]^−^ (Δ ppm −0.2), along with its sodium adduct at *m*/*z* 303.2293 [FA(18:2) + Na]^+^ (Δ ppm 0.6) and disodium adduct at *m*/*z* 325.2113 [FA(18:2) − H + 2Na]^+^ (Δ ppm −0.4), and oleic acid (C_18_H_34_O_2_) at m/z 281.2487 [FA(18:1) − H]^–^ (Δ ppm −0.3), along with its disodium adduct at *m*/*z* 327.2269 [FA(18:1) − H + 2Na]^+^ (Δ ppm −0.4). Weak signals for lauric and myristic acid were also observed. This preliminary assignment to fatty acids is confirmed by previous research work which successfully proved the anti-diabetic, AChE/BChE inhibiting and antibacterial effects for individual fatty acids [62,63,64,65]. Until now, apolar compounds such as fatty acids and triacylglycerols have often been overlooked in the *in vitro* assay analysis, as a defattening step is often carried out at the beginning of the workflow or such apolar compounds are hardly soluble in the buffered polar assay medium. However, it was clearly shown in this study that apolar components of a sample should not be discriminated by analytical methodologies and protocols as these were most active [62,63,64,65].

## 3. Materials and Methods

### 3.1. Reagents and Chemicals

All the chemicals and solvents used were analytical grade. Solvents used for HPTLC study were of chromatography grade. DPPH•, dimethyl sulfoxide, phosphate buffer, and tris (hydroxymethyl)aminomethane hydrochloride (Tris-HCl) buffer, methanol, ethanol, *n*-hexane, dichloromethane, ethyl acetate, *n*-butanol, 4-methoxybenzaldehyde, gallic acid, quercetin, potassium ferricyanide, trichloroacetic acid, distilled water, iron(III)chloride_,_ Folin–Ciocalteu reagent, sodium carbonate_,_ copper (II) chloride, neocuproin, ammonium acetate, electric eel (*Electrophorus electricus*) AChE (Type-VI-S, EC 3.1.1.7), equine serum BChE (EC 3.1.1.8), acetylthiocholine iodide and butyrylthiocholine chloride, 5,5′-dithio-bis(2-nitrobenzoic) acid, galantamine hydrobromide, rivastigmine, mushroom tyrosinase (EC 1.14.1.8.1; 30 U), *L*-DOPA, and α-kojic acid were purchased from Sigma (St. Louis, MO, USA). Acetic acid, bovine serum albumin, Dulbecco’s phosphate-buffered saline (DPBS), fluorescein-di-β-D-galactopyranoside (FDG), tetracycline, thiazol blue tetrazolium bromide, acarbose, α-amylase from hog pancreas; Gram’s iodine solution, 4-nitroquinoline-1-oxide was purchased from TCI (Eschborn, Germany). The bacteria, *i.e*., *Aliivibrio fischeri* (NRRI–B11177, strain 7151) and *Bacillus subtilis* (DSM-618) were purchased from the German Collection of Microorganisms and Cell Cultures (Leibniz Institute DSMZ, Berlin, Germany). *Salmonella enterica* subspecies *enterica* Typhimurium strain TA1535 (*Salmonella* Typhimurium), genetically modified to contain the plasmid pSK1002, was obtained from Trinova Biochem (Giessen, Germany). HPTLC plates silica gel 60 F_254_ and HPTLC plates silica gel 60, both 20 cm × 10 cm, were provided by Merck (Darmstadt, Germany), if necessary, prewashed with methanol–water (4:1) and followed by drying at 120 °C for 15 min (oven or TLC Plate Heater, CAMAG, Muttenz, Switzerland).

### 3.2. Origin and Lyophilization of Plant Materials

Samples of *Launaea taraxacifolia* (herbarium number 113627, Asteraceae), *Crassocephalum rubens* (herbarium number 113720, Asteraceae)*, Solanecio biafrae* (herbarium number 113776, Asteraceae), and *Basella alba* (herbarium number 113741, Basellaceae) were collected from the forest and vegetable center of the International Institute of Tropical Agriculture (Ibadan, Nigeria), while samples of *Solanum macrocarpon* (herbarium number 113641, Solanaceae) and *Gnetum africanum* (herbarium number 113862, Gnetaceae) were purchased from the local markets in Ibadan, Nigeria, in July 2021. The market plants were authenticated at Forest Herbarium Ibadan, Nigeria, after which they were freeze-dried using a lyophilizer (Labconco, Kansas, MO, USA), ground to powder, and stored in air-tight containers until use.

### 3.3. In Vitro Biological Assays after Extraction/Fractionation

#### 3.3.1. Extraction and Fractionation of Plant Powders

For ethanol and water extracts, the lyophilized plant powders (200 g each) were macerated separately in 2 L 96% ethanol or distilled water for 72 h, filtered, and concentrated in vacuo. The maceration was repeated 4 times until the filtrate color became light to achieve an exhaustive extraction. Depending on the yield, the ethanol extracts were dissolved in methanol–water 9:1, *V/V* (dry extract to solvent ratio of 1:2, *w/V*), which was fractionated in a separating funnel using four different solvents of increasing polarity (100 mL aliquots) each of *n*-hexane, dichloromethane, ethyl acetate, and *n*-butanol.

#### 3.3.2. TPC

Using the Folin–Ciocalteu method [25], the TPC was expressed as mg gallic acid equivalent per gram of plant extract. It was calculated from the gallic acid (mg GAE/g) calibration curve (y = 1.8829x + 0.1891, R^2^ = 0.9747) prepared by mixing 10 µL of gallic acid dilution (0.016–2 mg/mL in ethanol) or 10 µL plant extract/fraction (2 mg/mL dissolved in ethanol, 96%) with 30 μL of Folin–Ciocalteu reagent (two-fold dilution) and 150 μL of sodium carbonate (3.5% in water) in 96-microtiter plate wells, followed by incubation at 40 °C for 30 min. Absorbance was measured at 765 nm (microplate reader SpectraMax^®^ ABS Plus, Molecular Devices, San Jose, CA, USA).

#### 3.3.3. DPPH• Scavenging Assay

As described in [62,63,64], the plant extract/fraction (10 µL, 2 mg/mL) and references (gallic acid and quercetin, 2 mg/mL), all dissolved in ethanol, were mixed with ethanolic DPPH• solution (90 µL, 0.138 mg/mL) and incubated in the dark at room temperature for 30 min. The remaining DPPH• signal was measured at 515 nm using the microplate reader.

#### 3.3.4. FRAP Assay

According to Deniz et al. [66], 10 µL plant extract/fraction (2 mg/mL in ethanol) were added into 25 μL of phosphate buffer (pH 6.6) and 25 μL of potassium ferricyanide (1%, *w*/*v*), incubated at 50 °C for 20 min, and then 25 μL trichloroacetic acid (10%), 85 μL distilled water, and 17 μL iron(III)chloride (0.1%, *w*/*v*) were added. After 30 min incubation at room temperature, absorbance was read at 700 nm using the microplate reader.

#### 3.3.5. CUPRAC Assay

Following the method of Deniz et al. [66], 25 μL of 10 mM copper (II) chloride, 25 μL of 7.5 mM neocuproin, 25 μL of 1 M ammonium acetate (pH 7), 25 μL of plant extract/fraction (2 mg/mL) or reference gallic acid (2 mg/mL), and 100 μL of distilled water were mixed in a 96-well microtiter plate well. The reaction was incubated at room temperature for 30 min, after which the absorbance was measured at 450 nm using the microplate reader.

#### 3.3.6. AChE and BChE Inhibition Assays

Following Ellman’s spectrophotometric method [30] modified by [31], 140 µL of 0.1 mM sodium phosphate buffer (pH 8.0) was added to the 96-well microtiter plate, and then 20 µL of plant extract/fraction (2 mg/mL) as well as solvent blank (ethanol, negative control) was added. Afterwards, 20 µL of 0.2 M AChE/BChE solution (0.003 U/well) was added followed by incubation at room temperature for 10 min. The reaction was started by adding 10 μL of 0.2 M acetylthiocholine iodide/butyrylthiocholine chloride as respective substrate and, finally, 10 μL of 5,5′-dithio-bis(2-nitrobenzoic) acid. The formation rate and color intensity of the yellow-colored 2-nitro-5-thiobenzoate formed were measured using the microplate reader at wavelength of 412 nm. The positive control galantamine hydrobromide was used as the reference in both experiments. Enzyme activity was calculated by rate of change in absorbance by kinetics measurement every 1 min for 10 min using Softmax Pro Software for Windows 10 version 7 (San Jose, CA, USA). Percentage inhibition was calculated based on a comparison of rates of enzyme reaction between samples and the blank sample (ethanol in phosphate buffer, pH 8) using the formula (1 − S/E) ×100, where E is enzyme activity without test sample and S is enzyme activity with test sample.

#### 3.3.7. TYR Inhibition Assay

Inhibition of TYR was determined using L-DOPA as substrate [67], modified by Orhan et al. [68]. Plant extract/fraction (10 µL) dissolved in dimethyl sulfoxide were added to 80 μL of phosphate buffer (pH 6.8) in a 96-well plate and 30 µL of 5 mM *L*-DOPA, which was followed by 10 min incubation at 37 °C. Tyrosinase (1.2 U/well, 30 µL) was then added, the reaction was incubated at 25 °C for 20 min, and absorbance was measured at 492 nm using the microplate reader. Results were compared with the positive control α-kojic acid used as reference and the negative control dimethyl sulfoxide.

#### 3.3.8. Statistical Analysis

Antioxidant activity and enzyme inhibition assays were performed in triplicate or in four replicates, respectively. Values were expressed as means ± standard deviation (S.D). Correlation analysis was performed using Excel to determine Pearson’s correlation coefficient (r^2^). IC_50_ values were calculated for samples with minimum of 50% activity using GraphPad Prism 6.01. Significant level used was determined at *p* ≤ 0.05 from one-way ANOVA followed by Tukey’s multiple comparison test for the comparison of positive control with the test groups using GraphPad Prism 6.01. 

### 3.4. HPTLC–Bioactivity Profiling

#### 3.4.1. Extraction and HPTLC Analysis

Lyophilized plant powder samples (100 mg each) were weighed accurately, and each was dissolved in 2 mL ethyl acetate–ethanol–water 1:1:1, *V*/*V*/*V,* ultrasonicated for 30 min (Sonorex Digiplus, Bandelin, Berlin, Germany), and centrifuged at 3000× *g* for 15 min (Labofuge 400, Heraeus, Hanau, Germany). Each supernatant (50 mg/mL) was transferred to an autosampler vial. The plant extracts (5 µL/band, if not stated otherwise) were applied (ATS 4, CAMAG) on HPTLC plates silica gel 60 F_254_ (without F_254_ indicator for SOS-Umu-C bioassay), separated with ethyl acetate–toluene–methanol–water 4:1:1:0.4, *V*/*V*/*V*/*V,* or toluene–ethyl acetate 7:3, *V*/*V,* up to a migration distance of 70 mm (about 20 min, Twin Trough Chamber, CAMAG) and detected under white light illumination (reflection and transmission mode), UV 254 nm, and FLD 366 nm (TLC Visualizer, CAMAG). Immersion (3 cm/s, 2 s, TLC Immersion Device, CAMAG) or piezoelectric spraying (blue nozzle, level 3, Derivatizer, CAMAG) was used for the effect-directed assays as follows.

#### 3.4.2. HPTLC–DPPH• Scavenging Assay

The HPTLC chromatogram was sprayed with 4 mL DPPH• solution (0.04% in methanol), dried, and detected instantly and after one day under white light illumination as described by Morlock et al. [20]. Gallic acid (0.25 mg/mL in methanol; 0.2, 0.6, and 1.0 µL/band) was used as positive control. Antioxidant compounds were directly observed as yellow zones against a purple background. Detection was also performed a day later.

#### 3.4.3. HPTLC–AChE/BChE Inhibition Assays

Enzyme solution (3.0 mL, 6.66 U/mL AChE or 3.34 U/mL BChE in Tris–HCl buffer plus 1 mg bovine serum albumin) was sprayed on the HPTLC chromatogram, followed by incubation at 37 °C for 25 min, and then 0.5 mL substrate (3 mg/mL 1-naphthyl acetate solution in ethanol and Fast Blue B salt solution in water) was sprayed. The plates were dried and detected under white light illumination [21]. Rivastigmine (0.1 mg/mL in methanol; 2, 4, and 8 µL/band) was used as positive control. For both assays, inhibition zones were detected as colorless (white) zones on a purple background under white light illumination.

#### 3.4.4. HPTLC–α-Amylase/α-Glucosidase Inhibition Assays

According to Schreiner et al. [21], α-amylase solution (62.5 U/mL in sodium acetate buffer, pH 7) was sprayed on the HPTLC chromatogram, followed by incubation at 37 °C for 30 min and spraying of the substrate solution (2% starch in water), which was followed by another incubation at 37 °C for 20 min and spraying of Gram’s iodine solution (0.5 mL). For the α-glucosidase inhibition assay, 2-naphthyl-α-D-glucopyranoside substrate solution (2 mL, 12 mg in 10 mL ethanol with 10% 10 mM sodium chloride solution) was sprayed, and after drying for 2 min, α-glucosidase solution (2.5 mL, 10 U/mL in sodium acetate buffer, pH 7.5) was sprayed, followed by incubation at 37 °C for 15 min and spraying of Fast Blue B salt solution (0.75 mL, 2 mg/mL in water). Acarbose (0.01 mg/mL in methanol; 0.3, 0.6, and 0.9 μL/band) was used as positive control. For both assays, inhibition zones were detected as colorless (white) zones on a purple background under white light illumination.

#### 3.4.5. HPTLC–*Aliivibrio fischeri* Bioassay, Followed by Derivatization with *p*-Anisaldehyde Sulfuric Acid Reagent

The bioluminescent *Aliivibrio fischeri* suspension (evaluated upon shaking in a dark room) was sprayed on the HPTLC chromatogram, and the humid plate was transferred to the BioLuminizer (CAMAG) as described by Jamshidi-Aidji and Morlock [69]. Ten images of the bioluminescence were recorded over 30 min (exposure time 60 s, trigger interval 3.0 min), depicted as greyscale image. Dark zones revealed lower energetic metabolism of the bacteria, whereas bright zones indicated a higher energetic metabolism. As positive control, caffeine was used (1 mg/mL in methanol; 0.5, 1.5, and 3 μL/band).

The dried bioautogram was additionally derivatized by immersion in *p*-anisaldehyde sulfuric acid reagent (0.25 mL 4-methoxybenzaldehyde, 2 mL sulfuric acid, 4 mL glacial acetic acid, and 35 mL methanol) at 3 cm/s immersion speed for 2 s (TLC Immersion Device, CAMAG), followed by plate heating at 110 °C for 3 to 5 min (TLC Plate Heater, CAMAG) and detections under white light illumination and FLD 366 nm.

#### 3.4.6. HPTLC–*Bacillus subtilis* Bioassay

The bacterial *Bacillus subtilis* suspension, *i.e*., 100 µL cryostock in 20 mL 2.3% Müller–Hinton broth incubated overnight at 37 °C and adjusted to an optical density at 600 nm (OD_600_) of 1.0, was sprayed on the HPTLC chromatogram, followed by incubation at 37 °C for 2 h. Then, thiazol blue tetrazolium bromide substrate solution (0.2% in DPBS buffer) was sprayed on it, followed by incubation at 37 °C for 1.5 h, plate drying (50 °C, 10 min), and detection of colorless (white) antibacterial zones on a purple background under white light illumination [21]. As positive control, tetracycline (0.005 mg/mL in ethanol; 0.5, 1.5, and 3 µL/band) was used.

#### 3.4.7. HPTLC–SOS-Umu-C Genotoxicity Bioassay

The bioassay was performed on HPTLC plates without F_254_ according to [56,70]. The *Salmonella* Typhimurium suspension (OD_600_ of 0.2) was sprayed (2.8 mL) on the HPTLC chromatogram, followed by incubation at 37 °C for 3 h. In the case of S9 metabolization, S9-mix (500 µL) and the solutions of NADP (166 µL), G6P (42 µL), and buffer salts (953 µL) were added to the *Salmonella* Typhimurium suspension (3334 µL) as described [24]. FDG substrate solution (25 µL of 0.5% FDG in dimethyl sulfoxide in 2.5 mL phosphate buffer) was sprayed (2.5 mL), followed by incubation at 37 °C for 15 min, plate drying, and detection at 254 nm. As positive control, 4-nitroquinoline-1-oxide (1 ng/mL in methanol; 0.2, 0.5, and 1 µL/band) and aflatoxin B1 (1 ng/µL in methanol; 1, 2.5, and 5 µL/band) were used without and with S9 metabolization, respectively. Genotoxic substances appeared as bright green fluorescent fluorescein zones (released from FDG via β-galactosidase produced by the bacteria in the presence of DNA-damaging compounds) on a less green fluorescent background at FLD 254 nm.

#### 3.4.8. HPTLC–Cytotoxicity Bioassay

The *Salmonella* Typhimurium suspension (OD_600_ of 0.2) was sprayed (2.8 mL) on the HPTLC chromatogram. Thiazol blue tetrazolium bromide substrate solution (0.2% in phosphate buffer) was sprayed (800 μL) onto the still-wet HPTLC chromatogram. The plate was then incubated (37 °C, 18 h), followed by plate drying in a stream of cold air and detection under white light illumination [23].

#### 3.4.9. HPTLC–UV/Vis/FLD–HESI–HRMS

*Basella alba* (5 µL/band) was applied exemplarily. After separation using the apolar mobile phase system (toluene–ethyl acetate 7:3, *V/V*), the bioactive zone 1 was eluted with methanol containing 0.1% formic acid at a flow rate of 100 µL/s via the open-source modified auto-TLC-LC-MS interface [61] from the HPTLC plate into the HESI–HRMS system (QExactive Plus, Thermo Fisher Scientific, Dreieich, Germany) with the following settings: +3.5 kV and −3.5 kV spray voltage, 270 °C capillary and 200 °C probe heater temperature, resolution 280,000, *m*/*z* 100–1500, and automatic maximum injection time 10/200 ms for positive/negative ionization. The MZMine3 peak picking software was used [71]. After the zone elution, the respective α-amylase inhibition assay (3.4.4.) was performed to confirm the proper positioning on the active zone.

## 4. Conclusions

The bioactivity data for the six African under-utilized medicinal leafy vegetables *B. alba*, *C. rubens, G. africanum*, *L. taraxacifolia, S. biafrae*, and *S. macrocarpon* obtained by two different effect-directed analysis techniques, *i.e*., *in vitro* microtiter plate assays and HPTLC–bioactivity profiling, were consistent and in accordance with the sparse literature. Among the six leafy vegetables, *C. rubens, S. biafrae*, and *S. macrocarpon* in particular showed pronounced compounds that were antioxidative, antibacterial against Gram-positive *Bacillus subtilis* and Gram-negative *Aliivibrio fischeri*, and inhibited AChE, BChE, α-amylase, and α-glucosidase. However, cytotoxic and genotoxic compounds were also observed. Altogether, the antioxidant, antimicrobial, antidiabetic, anticholinesterase, cytotoxic, and genotoxic activities may explain the traditional use of the plant material and validate some of their ethnomedicinal uses in the treatment of diabetes and in wound healing. Such leafy vegetables with health promoting benefits can serve for the development of important local functional foods. Further work is ongoing to isolate and characterize the bioactive compounds from *C. rubens, S. biafrae*, and *S. macrocarpon*.

## Figures and Tables

**Table 1:** Total phenolic content (TPC), ferric reducing antioxidant power (FRAP), cupric reduction antioxidant capacity (CUPRAC), and 2,2-diphenyl-1-picrylhydrazyl (DPPH•) scavenging activity of *Basella alba* (BA), *Crassocephalum, rubens* (CR), *Gnetum africanum* (GA), *Launea taraxacifolia* (LT), *Solanecio biafrae* (SB), and *Solanum macrocarpon* (SM) extracts/fractions; ethanol EtOH, dichloromethane DCM, ethyl acetate EtOAc, 9:1 mixture of methanol MeOH and water H_2_O; in bold are highest values discussed.

Plant	Extract/Fraction	TPC (mg GAE/g) ±S.D. ^b^	FRAP at 700 nm ^a^ Absorbance ± S.D. ^b^ at 107 µg/mL	CUPRAC at 450 nm ^a^ Absorbance ± S.D. ^b^ at 250 µg/mL)	DPPH• Activity (% ± S.D. ^b^ at 200 µg/mL) IC_50_ (µg/mL)
BA	EtOH	5.4 ± 0.7	0.78 ± 0.07	0.72 ± 0.03	19.2 ± 0.6
	*n*-Hexane	4.0 ± 0.6	0.79 ± 0.09	0.83 ± 0.05	19.9 ± 2.7
	DCM	4.6 ± 0.4	0.95 ± 0.14	0.96 ± 0.03	14.1 ± 4.3
	EtOAc	17.3 ± 0.8	0.77 ± 0.05	0.72 ± 0.06	26.9 ± 3.0
	*n*-Butanol	17.0 ± 0.4	0.78 ± 0.03	0.58 ± 0.03	17.0 ± 2.2
	MeOH-H_2_O	NA	0.67 ± 0.03	0.16 ± 0.00	4.8 ± 0.4
	H_2_O	NA	0.77 ± 0.03	ND	NA
CR	EtOH	23.3 ± 1.3	1.29 ± 0.04 **	1.89 ± 0.02 ***	64.7 ± 5.8 * IC_50_ 157.8 ± 27.7
	*n*-Hexane	5.9 ± 0.5	0.84 ± 0.12	0.99 ± 0.04	17.4 ± 3.0
	DCM	10.2 ± 0.4	0.83 ± 0.11	1.33 ± 0.14	22.5 ± 4.6
	EtOAc	38.7 ± 3.2 ***	1.83 ± 0.04 ****	1.96 ± 0.02 ***	87.2 ± 0.5 *** **IC_50_ 27.7 ± 4.5**
	*n*-Butanol	40.2 ± 1.0 ***	1.69 ± 0.01 ***	1.69 ± 0.04 ***	84.7 ± 0.4 *** **IC_50_ 47.7 ± 7.8**
	MeOH-H_2_O	7.1 ± 0.3	0.97 ± 0.05	0.87 ± 0.02	28.9 ± 4.2
	H_2_O	7.4 ± 0.1	0.75 ± 0.20	ND	NA
GA	EtOH	10.2 ± 1.1	0.73 ± 0.09	0.93 ± 0.15	18.5 ± 1.5
	*n*-Hexane	9.6 ± 0.7	0.86 ± 0.06	1.16 ± 0.10	30.6 ± 1.2
	DCM	22.2 ± 0.6	0.98 ± 0.06	1.62 ± 0.07 ***	37.2 ± 4.9
	EtOAc	21.6 ± 1.6	0.99 ± 0.03	1.12 ± 0.05	45.6 ± 5.2
	*n*-Butanol	5.9 ± 0.1	0.72 ± 0.08	0.50 ± 0.00	16.1 ± 2.7
	MeOH-H_2_O	NA	0.70 ± 0.06	0.18 ± 0.00	12.2 ± 2.4
	H_2_O	8.1 ± 0.4	0.82 ± 0.03	ND	10.7 ± 2.4
LT	EtOH	3.3 ± 0.4	0.70 ± 0.01	0.90 ± 0.03	9.4 ± 1.5
	*n*-Hexane	5.2 ± 0.6	0.79 ± 0.06	0.97 ± 0.06	8.6 ± 3.9
	DCM	3.6 ± 0.5	0.81 ± 0.03	0.74 ± 0.03	14.0 ± 2.1
	EtOAc	12.6 ± 0.6	0.97 ± 0.11	1.16 ± 0.02	38.5 ± 1.3
	*n*-Butanol	2.6 ± 0.4	0.71 ± 0.04	0.62 ± 0.03	13.6 ± 1.6
	MeOH-H_2_O	NA	0.74 ± 0.03	0.31 ± 0.01	13.6 ± 2.4
	H_2_O	12.5 ± 1.0	1.04 ± 0.13	ND	52.0 ± 3.6 IC_50_ 201.8 ± 14.3
SB	EtOH	8.6 ± 1.1	0.84 ± 0.04	1.22 ± 0.06	27.0 ± 1.9
	*n*-Hexane	6.7 ± 0.8	0.76 ± 0.10	1.02 ± 0.07	5.4 ± 1.6
	DCM	7.9 ± 0.4	0.85 ± 0.05	1.18 ± 0.06	9.9 ± 1.5
	EtOAc	31.0 ± 4.0 *	1.56 ± 0.04 ***	1.72 ± 0.05 ***	84.4 ± 1.1 *** **IC_50_ 62.8 ± 2.2**
	*n*-Butanol	13.5 ± 0.4	1.00 ± 0.03	1.03 ± 0.06	32.5 ± 2.4
	MeOH-H_2_O	NA	0.77 ± 0.04	0.31 ± 0.00	45.1 ± 3.6
	H_2_O	1.0 ± 0.1	0.82 ± 0.06	ND	15.9 ± 4.4
SM	EtOH	15.2 ± 2.0	1.15 ± 0.03 **	1.26 ± 0.14	78.9 ± 2.3 ** **IC_50_ 62.7 ± 4.4**
	*n*-Hexane	4.2 ± 0.4	0.92 ± 0.02	0.79 ± 0.01	36.6 ± 0.6
	DCM	8.4 ± 1.1	0.93 ± 0.03	1.08 ± 0.03	51.2 ± 2.0 IC_50_ 313.5 ± 11.4
	EtOAc	20.0 ± 1.7	1.11 ± 0.02 **	1.51 ± 0.04 ***	52.6 ± 2.4 IC_50_ 186.4 ± 3.3
	*n*-Butanol	34.9 ± 2.0 **	1.61 ± 0.09 ***	1.70 ± 0.05 ***	84.9 ± 2.0 *** **IC_50_ 73.5 ± 3.9**
	MeOH-H_2_O	4.8 ± 1.2	0.90 ± 0.02	0.74 ± 0.01	64.4 ± 2.4 * IC_50_ 84.1 ± 7.5
	H_2_O	1.2 ± 0.2	0.79 ± 0.01	ND	NA
References	Quercetin (200 µg/mL)		2.01 ± 0.03 ****		89.5 ± 0.3 *** **IC_50_ 6.7 ± 0.8**
	Gallic acid (100 µg/mL)			2.85 ± 0.55 ****	

^a^ Higher absorbance indicates higher antioxidant activity in FRAP and CUPRAC; ^b^ standard deviation (*n* = 3); activity in comparison with reference: * *p* < 0.05, ** *p* < 0.01, *** *p* < 0.001, **** *p* < 0.0001; NA: no activity at tested concentration; ND: activity not determined.

**Table 2:** Butylrylcholinesterase (BChE), acetylcholinesterase (AChE), and tyrosinase (TYR) inhibitory activities of *Basella alba* (BA), *Crassocephalum, rubens* (CR), *Gnetum africanum* (GA), *Launea taraxacifolia* (LT), *Solanecio biafrae* (SB), and *Solanum macrocarpon* (SM); in bold are highest values discussed.

Plants	Extract/Fraction	BChE Inhibition (% ± S.D. ^a^ at 200 µg/mL) IC_50_ (µg/mL)	AChE Inhibition (% ± S.D. ^a^ at 200 µg/mL) IC_50_ (µg/mL)	TYR Inhibition (% ± S.D. ^a^ at 667 µg/mL) IC_50_ not determined
BA	EtOH	53.0 ± 3.6 * **IC_50_ 121.5 ± 23.5**	10.4 ± 2.7	NA
	*n*-Hexane	34.7 ± 0.8	12.1 ± 1.8	NA
	DCM	14.1 ± 0.7	17.6 ± 2.1	NA
	EtOAc	15.1 ± 5.0	11.3 ± 1.3	7.9 ± 2.0
	*n*-Butanol	23.6 ± 1.9	5.3 ± 0.6	5.3 ± 1.5
	MeOH-H_2_O	22.9 ± 3.4	13.5 ± 4.0	3.3 ± 0.7
	H_2_O	36.3 ± 3.6	NA	6.2 ± 0.1
CR	EtOH	20.7 ± 1.8	29.8 ± 3.7	8.5 ± 0.8
	*n*-Hexane	25.7 ± 6.6	12.0 ± 3.8	NA
	DCM	4.9 ± 0.9	16.1 ± 2.9	NA
	EtOAc	20.6 ± 5.9	13.7 ± 0.9	27.9 ± 1.0
	*n*-Butanol	19.2 ± 5.8	17.8 ± 4.1	42.9 ± 0.2 **
	MeOH-H_2_O	52.0 ± 2.4 * **IC_50_ 176.8 ± 13.6**	NA	7.6 ± 0.7
	H_2_O	29.2 ± 3.7	NA	NA
GA	EtOH	18.6 ± 1.5	13.3 ± 3.0	10.2 ± 1.4
	*n*-Hexane	28.8 ± 7.1	20.0 ± 6.2	NA
	DCM	NA	23.8 ± 4.1	NA
	EtOAc	15.4 ± 4.4	NA	33.9 ± 3.7 *
	*n*-Butanol	24.3 ± 1.6	18.3 ± 3.2	8.7 ± 0.2
	MeOH-H_2_O	32.2 ± 0.1	13.9 ± 1.7	5.1 ± 1.5
	H_2_O	16.5 ± 1.6	NA	10.7 ± 2.4
LT	EtOH	30.1 ± 3.5	11.5 ± 2.9	NA
	*n*-Hexane	34.3 ± 2.6	24.0 ± 2.4	NA
	DCM	NA	22.9 ± 0.2	2.1 ± 0.1
	EtOAc	NA	25.7 ± 3.7	20.7 ± 3.7
	*n*-Butanol	13.8 ± 2.1	19.6 ± 1.6	12.3 ± 3.0
	MeOH-H_2_O	26.9 ± 2.8	25.7 ± 2.5	7.0 ± 3.2
	H_2_O	66.4 ± 6.4 *** **IC_50_ 114.0 ± 24.2**	30.4 ± 2.2	3.6 ± 0.1
SB	EtOH	44.6 ± 0.9*	23.5 ± 0.5	NA
	*n*-Hexane	39.1 ± 0.7	26.5 ± 2.9	NA
	DCM	NA	28.2 ± 1.2	NA
	EtOAc	13.8 ± 1.7	16.7 ± 2.3	37.4 ± 0.7 *
	*n*-Butanol	28.6 ± 4.4	NA	27.0 ± 2.1
	MeOH-H_2_O	58.8 ± 4.1 ** **IC_50_ 135.3 ± 16.6**	53.6 ± 3.8 * **IC_50_ 207.0 ± 26.2**	8.2 ± 1.8
	H_2_O	52.6 ± 4.7 * **IC_50_ 183.4 ± 26.3**	30.7 ± 4.8	7.4 ± 2.7
SM	EtOH	19.6 ± 6.0	22.4 ± 1.7	15.2 ± 1.3
	*n*-Hexane	9.9 ± 2.0	21.1 ± 3.3	NA
	DCM	NA	30.8 ± 1.4	NA
	EtOAc	24.8 ± 2.1	38.2 ± 0.3	17.4 ± 1.2
	*n*-Butanol	NA	30.4 ± 1.6	18.2 ± 2.0
	MeOH-H_2_O	36.1 ± 3.2	18.5 ± 0.9	13.3 ± 2.9
	H_2_O	35.7 ± 2.2	20.2 ± 2.9	3.3 ± 0.5
References	Galantamine (50 µg/mL)		97.8 ± 0.1 **** **IC_50_ 0.8 ± 0.1**	
	Galantamine (200 µg/mL)	69.6 ± 1.7 *** **IC_50_ 112.2 ± 9.7**		
	α-Kojic acid (500 µg/mL)			87.5 ± 1.2 ***

^a^ Standard deviation (*n* = 4); activity in comparison with control: * *p* < 0.05, ** *p* < 0.01, *** *p* < 0.001, **** *p* < 0.0001; NA: no activity at tested concentration.

## Data Availability

The data presented in this study are available on request from the corresponding author. The data are not publicly available due to instrument software compatibility.

## References

[1] Gurib-Fakim A. (2017). Capitalize on African biodiversity. Nature.

[2] Sosef M.S., Dauby G., Blach-Overgaard A., van der Burgt X., Catarino L., Damen T., Deblauwe V., Dessein S., Dransfield J., Droissart V. (2017). Exploring the floristic diversity of tropical Africa. BMC Biol..

[3] Ayodele A. (2005). The Medicinally important leafy vegetables of Southwestern Nigeria. Ethnobot. Leafl..

[4] Ogunrotimi D., Kayode J., Odesola F. (2018). Ethnobotany and conservation of indigenous vegetables in Ekiti State, Nigeria. Singapore J. Sci. Res..

[5] Deshmukh S., Gaikwad D. (2014). A review of the taxonomy, ethnobotany, phytochemistry and pharmacology of *Basella alba* (Basellaceae). J. Appl. Pharm. Sci..

[6] Iweala E.E.J., Ogidigo J.O. (2015). Prostate specific antigen, antioxidant and hematological parameters in prostatic rats fed *Solanum macrocarpon* L. leaves. Asian J. Biol. Sci..

[7] Omoregie E.S., Okugbo O.T., Oikeh E., Irabor F. (2015). Hepatoprotective effect of leaf extracts of *Crassocephalum rubens* (Juss. ex Jacq.) S. Moore in rifampicin-induced oxidative stress in Swiss mice. J. Pharm. Biores..

[8] Bello O.M., Zaki A.A., Khan S., Fasinu P.S., Ali Z., Khan I.A., Usman L., Oguntoye O. (2017). Assessment of selected medicinal plants indigenous to West Africa for antiprotozoal activity. S. Afr. J. Bot..

[9] Bello O.A., Ayanda O.I., Aworunse O.S., Olukanmi B.I., Soladoye M.O., Esan E.B., Obembe O.O. (2018). *Solanecio biafrae*: An underutilized nutraceutically-important African indigenous vegetable. Pharmacogn. Rev..

[10] Ayuk E., Oforji C.F., Ugwu F., Aronimo S.B., Njokunwogbu A. (2017). Determination of secondary metabolites and biological potential of *Gnetun africanum* (Okazi) Leaves. Pharm. Chem. J..

[11] Harvey A.L. (2008). Natural products in drug discovery. Drug Discov. Today.

[12] Choudhary M.I., Thomsen W.J. (2001). Bioassay Techniques for Drug Development.

[13] Yang X., Wei H.M., Hu G.Y., Zhao J., Long L.N., Li C.J., Zhao Z.J., Zeng H.K., Nie H. (2020). Combining antioxidant astaxantin and cholinesterase inhibitor huperzine A boosts neuroprotection. Mol. Med. Rep..

[14] Collins A.E., Saleh T.M., Kalisch B.E. (2022). Naturally occurring antioxidant therapy in Alzheimer’s disease. Antioxidants.

[15] Agatonovic-Kustrin S., Kettle C., Morton D.W. (2018). A molecular approach in drug development for Alzheimer’s disease. Biomed. Pharmacother..

[16] Huat T.J., Camats-Perna J., Newcombe E.A., Valmas N., Kitazawa M., Medeiros R. (2019). Metal toxicity links to Alzheimer’s disease and neuroinflammation. J. Mol. Biol..

[17] Nagatsu T., Nakashima A., Watanabe H., Ito S., Wakamatsu K., Zucca F.A., Zecca L., Youdim M., Wulf M., Riederer P. (2023). The role of tyrosine hydroxylase as a key player in neuromelanin synthesis and the association of neuromelanin with Parkinson’s disease. J. Neural Transm..

[18] Carballo-Carbajal I., Laguna A., Romero-Giménez J., Cuadros T., Bové J., Martinez-Vicente M., Parent A., Gonzalez-Sepulveda M., Peñuelas N., Torra A. (2019). Brain tyrosinase overexpression implicates age-dependent neuromelanin production in Parkinson’s disease pathogenesis. Nat. Commun..

[19] Morlock G.E. (2022). Planar chromatographic super-hyphenations for rapid dereplication. Phytochem. Rev..

[20] Morlock G.E., Heil J., Inarejos-Garcia A.M., Maeder J. (2021). Effect-directed profiling of powdered tea extracts for catechins, theaflavins, flavonols and caffeine. Antioxidants.

[21] Schreiner T., Sauter D., Friz M., Heil J., Morlock G.E. (2021). Is our natural food our homeostasis? Array of a thousand effect-directed profiles of 68 herbs and spices. Front. Pharmacol..

[22] Morlock G.E. (2014). Chromatography combined with bioassays and other hyphenations–the direct link to the compound indicating the effect. Instrumental Methods for the Analysis and Identification of Bioactive Molecules.

[23] Morlock G.E., Meyer D. (2023). Designed genotoxicity profiling detects genotoxic compounds in staple food such as healthy oils. Food Chem..

[24] Debon E., Rogeboz P., Latado H., Morlock G.E., Meyer D., Cottet-Fontannaz C., Scholz G., Schilter B., Marin-Kuan M. (2022). Incorporation of metabolic activation in the HPTLC-SOS-Umu-C bioassay to detect low levels of genotoxic chemicals in food contact materials. Toxics.

[25] Orhan I., Şenol F., Gülpinar A., Kartal M., Şekeroglu N., Deveci M., Kan Y., Şener B. (2009). Acetylcholinesterase inhibitory and antioxidant properties of *Cyclotrichium niveum*, *Thymus praecox* subsp. *caucasicus* var. *caucasicus*, *Echinacea purpurea* and *E. pallida*. Food Chem. Toxicol..

[26] Ferreira I.C., Baptista P., Vilas-Boas M., Barros L. (2007). Free-radical scavenging capacity and reducing power of wild edible mushrooms from northeast Portugal: Individual cap and stipe activity. Food Chem..

[27] Ribeiro J.P., Magalhaes L.M., Reis S., Lima J.L., Segundo M.A. (2011). High-throughput total cupric ion reducing antioxidant capacity of biological samples determined using flow injection analysis and microplate-based methods. Anal. Sci..

[28] Yusuff O., Omotosho K., Mustapha K., Abdulraheem A. (2020). Kinetics of the antioxidant activities of *Solanum macrocapon* and *Crassocephalum rubens* by DPPH radical scavenging method. J. Chem. Soc. Niger..

[29] Gouveia S., Castilho P.C. (2011). Antioxidant potential of *Artemisia argentea* L’Hér alcoholic extract and its relation with the phenolic composition. Food Res. Int..

[30] Ellman G.L., Courtney K.D., Andres Jr V., Featherstone R.M. (1961). A new and rapid colorimetric determination of acetylcholinesterase activity. Biochem. Pharmacol..

[31] Şenol F.S., Orhan I., Celep F., Kahraman A., Doğan M., Yilmaz G., Şener B. (2010). Survey of 55 Turkish *Salvia* taxa for their acetylcholinesterase inhibitory and antioxidant activities. Food Chem..

[32] Adedayo B. (2019). Effect of blanching on inhibition of cholinesterases and antioxidative properties of phenolic extracts of African lettuce (*Launaea taraxacifolia*). J. Appl. Sci. Environ. Manag..

[33] Oboh G., Busari G.M., Ademosun A.O., Oyeleye S.I. (2022). Effect of dietary inclusion of fireweed (*Crassocephalum crepidioides*) on behavioural patterns, memory indices, and activities of cholinergic and monoaminergic enzymes in a fruit fly (*Drosophila melanogaster*) model of Alzheimer’s disease. Food Front..

[34] Ajiboye B.O., Ojo O.A., Okesola M.A., Akinyemi A.J., Talabi J.Y., Idowu O.T., Fadaka A.O., Boligon A.A., Anraku de Campos M.M. (2018). *In vitro* antioxidant activities and inhibitory effects of phenolic extract of *Senecio biafrae* (Oliv and Hiern) against key enzymes linked with type II diabetes mellitus and Alzheimer’s disease. Food Sci. Nutr..

[35] Oboh G., Nwanna E.E., Oyeleye S.I., Olasehinde T.A., Ogunsuyi O.B., Boligon A.A. (2016). *In vitro* neuroprotective potentials of aqueous and methanol extracts from *Heinsia crinita* leaves. Food Sci. Hum. Wellness.

[36] Morlock G., Busso M., Tomeba S., Sighicelli A. (2021). Effect-directed profiling of 32 vanilla products, characterization of multi-potent compounds and quantification of vanillin and ethylvanillin. J. Chromatogr. A.

[37] Inarejos-Garcia A.M., Heil J., Martorell P., Álvarez B., Llopis S., Helbig I., Liu J., Quebbeman B., Nemeth T., Holmgren D. (2023). Effect-directed, chemical and taxonomic profiling of peppermint proprietary varieties and corresponding leaf extracts. Antioxidants.

[38] Krentz A.J., Bailey C.J. (2005). Oral antidiabetic agents: Current role in type 2 diabetes mellitus. Drugs.

[39] Adjatin A., Dansi A., Eze C., Assogba P., Dossou-Aminon I., Akpagana K., Akoègninou A., Sanni A. (2012). Ethnobotanical investigation and diversity of Gbolo (*Crassocephalum rubens* (Juss. ex Jacq.) S. Moore and *Crassocephalum crepidioides* (Benth.) S. Moore), a traditional leafy vegetable under domestication in Benin. Genet. Resour. Crop Evol..

[40] Sanoussi F., Ahissou H., Dansi M., Hounkonnou B., Agre P., Dansi A. (2015). Ethnobotanical investigation of three traditional leafy vegetables [*Alternanthera sessilis* (L.) DC., *Bidens pilosa* L., *Launaea taraxacifolia* Willd.] widely consumed in southern and central Benin. J. Biodivers. Environ. Sci..

[41] Ayoola M., Adebajo A., Zotor F., Pinkoane M. (2019). Justifying antidiabetic ethnomedicinal claim of *Senecio biafrae* through its antihyperglycemic and anti-oxidant activities. Ann. Complement. Altern. Med..

[42] Ajiboye B., Edobor G., Ojo A., Onikanni S., Olaranwaju O., Muhammad N. (2014). Effect of aqueous leaf extract of *Senecio biafrae* on hyperglycaemic and serum lipid profile of alloxan-induced diabetic rats. Int. J. Dis. Disord..

[43] Oyebode O.A., Erukainure O.L., Ibeji C., Koorbanally N.A., Islam M.S. (2019). *Crassocephalum rubens*, a leafy vegetable, suppresses oxidative pancreatic and hepatic injury and inhibits key enzymes linked to type 2 diabetes: An *ex vivo* and *in silico* study. J. Food Biochem..

[44] Gbadamosi I., Adeyi A., Oyekanmi O., Somade O. (2020). *Launaea taraxacifolia* leaf partitions ameliorate alloxan-induced pathophysiological complications *via* antioxidant mechanisms in diabetic rats. Metab. Open.

[45] Adjei D.-G.G., Mireku-Gyimah N.A., Sarkodie J.A., Nguessan B.B., Kodua E., Amedior J.K., Lartey I.A., Adi-Dako O., Asiedu-Gyekye I.J., Nyarko A.K. (2022). Antidiabetic properties of an ethanolic leaf extract of *Launaea taraxacifolia* (Willd.) Amin ex C. Jeffrey (Asteraceae) in SD rats. Clin. Phytosci..

[46] Azad A., Wan Azizi W., Babar Z., Labu Z.K., Zabin S. (2013). An overview on phytochemical, anti-inflammatory and anti-bacterial activity of *Basella alba* leaves extract. Middle East J. Sci. Res..

[47] Gbadamosi I., Alia A., Okolosi O. (2012). *In vitro* antimicrobial activities and nutritional assessment of roots of ten Nigerian vegetables. N. Y. Sci. J..

[48] Coker M., Ekpe I., Adewuyi O., Onu C. (2021). *In vitro* antimicrobial activity and bactericidal kinetics of the leaf extracts and fractions of *Gnetum africanum* on clinical wound isolates. Afr. J. Biomed. Res..

[49] Ilodibia C., Akachukwu E., Chukwuma M., Igboabuchi N., Adimonyemma R., Okeke N. (2016). Proximate, phytochemical and antimicrobial studies on *Solanum macrocarpon* L.. J. Adv. Biol. Biotechnol..

[50] Kumar B.R., Anupam A., Manchikanti P., Rameshbabu A.P., Dasgupta S., Dhara S. (2018). Identification and characterization of bioactive phenolic constituents, anti-proliferative, and anti-angiogenic activity of stem extracts of *Basella alba* and *rubra*. J. Food Sci. Technol..

[51] Alhassan S.O., Atawodi S.E.-O. (2019). Chemopreventive effect of dietary inclusion with *Crassocephalum rubens* (Juss ex Jacq) leaf on *N*-methyl-*N*-nitrosourea (MNU)-induced colorectal carcinogenesis in Wistar rats. J. Funct. Foods.

[52] Gadkari K., Kolhatkar U., Hemani R., Campanelli G., Cai Q., Kumar A., Levenson A.S. (2020). Therapeutic potential of gnetin C in prostate cancer: A pre-clinical study. Nutrients.

[53] Espinoza J.L., Inaoka P.T. (2017). Gnetin-C and other resveratrol oligomers with cancer chemopreventive potential. Ann. N. Y. Acad. Sci..

[54] Koukoui O., Agbangnan P., Boucherie S., Yovo M., Nusse O., Combettes L., Sohounhloué D. (2015). Phytochemical study and evaluation of cytotoxicity, antioxidant and hypolipidemic properties of *Launaea taraxacifolia* leaves extracts on cell lines HepG2 and PLB985. Am. J. Plant Sci..

[55] Oluremi B.B., Oloche J.J., Adeniji A.J. (2021). Anticancer and antibacterial activities of *Solanum aethiopicum* L., *Solanum macrocarpon* L. and *Garcinia kola* Heckel. Trop. J. Nat. Prod. Res..

[56] Meyer D., Marin-Kuan M., Debon E., Serrant P., Cottet-Fontannaz C., Schilter B., Morlock G.E. (2021). Detection of low levels of genotoxic compounds in food contact materials using an alternative HPTLC-SOS-Umu-C assay. ALTEX.

[57] Ayoola-Oresanya I.O., Sonibare M.A., Gueye B., Paliwal R., Abberton M.T., Morlock G.E. (2020). Effect-directed profiling and identification of bioactive metabolites from field, *in vitro*-grown and acclimatized *Musa* spp. accessions using high-performance thin-layer chromatography-mass spectrometry. J. Chromatogr. A.

[58] Galarce-Bustos O., Pavón-Pérez J., Henríquez-Aedo K., Aranda M. (2019). An improved method for a fast screening of α-glucosidase inhibitors in cherimoya fruit (*Annona cherimola* Mill.) applying effect-directed analysis *via* high-performance thin-layer chromatography-bioassay-mass spectrometry. J. Chromatogr. A.

[59] Morlock G.E., Koch J., Schwack W. (2023). Miniaturized open-source 2LabsToGo screening of lactose-free dairy products and saccharide-containing foods. J. Chromatogr. A.

[60] Sing L., Schwack W., Göttsche R., Morlock G.E. (2022). 2LabsToGo—Recipe for Building Your Own Chromatography Equipment Including Biological Assay and Effect Detection. Anal. Chem..

[61] Mehl A., Schwack W., Morlock G.E. (2021). On-surface autosampling for liquid chromatography−mass spectrometry. J. Chromatogr. A.

[62] Chandana N.G.A.S.S., Morlock G.E. (2021). Eight different bioactivity profiles of 40 cinnamons to discover multipotent compounds by multi-imaging planar chromatography hyphenated with effect-directed analysis and high-resolution mass spectrometry. Food Chem..

[63] Chandana N.G.A.S.S., Morlock G.E. (2021). Comprehensive bioanalytical multi-imaging by planar chromatography *in situ* combined with biological and biochemical assays highlights bioactive fatty acids in abelmosk. Talanta.

[64] Nikolaichuk H., Choma I.M., Morlock G.E. (2023). Bioactivity Profiles on 15 different effect mechanisms for 15 golden root products *via* high-performance thin-layer chromatography, planar assays, and high-resolution mass spectrometry. Molecules.

[65] Schreiner T., Eggerstorfer N.M., Morlock G.E. (2023). Ten-dimensional hyphenation including simulated static gastro-intestinal digestion on the adsorbent surface, planar assays, and bioactivity evaluation for meal replacement products. Food Funct..

[66] Deniz F.S.S., Orhan I.E., Duman H. (2021). Profiling cosmeceutical effects of various herbal extracts through elastase, collagenase, tyrosinase inhibitory and antioxidant assays. Phytochem. Lett..

[67] Masuda T., Yamashita D., Takeda Y., Yonemori S. (2005). Screening for tyrosinase inhibitors among extracts of seashore plants and identification of potent inhibitors from *Garcinia subelliptica*. Biosci. Biotechnol. Biochem..

[68] Orhan I.E., Tosun F., Deniz F.S.S., Eren G., Mıhoğlugil F., Akalgan D., Miski M. (2021). Butyrylcholinesterase-inhibiting natural coumarin molecules as potential leads. Phytochem. Lett..

[69] Jamshidi-Aidji M., Morlock G.E. (2016). From bioprofiling and characterization to bioquantification of natural antibiotics by direct bioautography linked to high-resolution mass spectrometry: Exemplarily shown for *Salvia miltiorrhiza* root. Anal. Chem..

[70] Meyer D., Marin-Kuan M., Mayrhofer E., Kirchnawy C., Debon E., Latado H., Patin A., Schilter B., Morlock G. (2023). Effect-detection by planar SOS-Umu-C genotoxicity bioassay and chemical identification of genotoxins in packaging migrates, proven by microtiter plate assays SOS-Umu-C and Ames-MPF. Food Control.

[71] Pluskal T., Castillo S., Villar-Briones A., Orešič M. (2010). MZmine 2: Modular framework for processing, visualizing, and analyzing mass spectrometry-based molecular profile data. BMC Bioinform..

